# Advancing the early detection of canine cognitive dysfunction syndrome with machine learning-enhanced blood-based biomarkers

**DOI:** 10.3389/fvets.2024.1390296

**Published:** 2024-08-07

**Authors:** Chae Young Kim, Jinhye Kim, Sunmi Yoon, Isaac Jinwon Yi, Hyuna Lee, Sanghyuk Seo, Dae Won Kim, Soohyun Ko, Sun-A Kim, Changhyuk Kwon, Sun Shin Yi

**Affiliations:** ^1^BK21 Four program, Department of Medical Sciences, Soonchunhyang University, Asan, Republic of Korea; ^2^iCONNECTOME, Co., Ltd., Cheonan, Republic of Korea; ^3^Department of Cognitive Science, University of California, San Diego, La Jolla, CA, United States; ^4^iamdt, Co., Ltd., Seoul, Republic of Korea; ^5^VIP Animal Medical Center, Seoul, Republic of Korea; ^6^Department of Biochemistry and Molecular Biology, Research Institute of Oral Sciences, College of Dentistry, Gangneung-Wonju National University, Gangneung, Republic of Korea; ^7^GenesisEgo, Co., Ltd., Seoul, Republic of Korea; ^8^Department of Clinical Sciences, College of Veterinary Medicine, Cornell University, Ithaca, NY, United States; ^9^Department of Biomedical Laboratory Science, Soonchunhyang University, Asan, Republic of Korea

**Keywords:** biomarker, blood–brain barrier, canine cognitive dysfunction syndrome, CCD rating scale, C-X-C chemokine ligand 10, NADPH oxidase 4, retinol binding protein 4, machine learning

## Abstract

Up to half of the senior dogs suffer from canine cognitive dysfunction syndrome (CCDS), the diagnosis method relies on subjective questionnaires such as canine cognitive dysfunction rating (CCDR) scores. Therefore, the necessity of objective diagnosis is emerging. Here, we developed blood-based biomarkers for CCDS early detection. Blood samples from dogs with CCDR scores above 25 were analyzed, and the biomarkers retinol-binding protein 4 (RBP4), C-X-C-motif chemokine ligand 10 (CXCL10), and NADPH oxidase 4 (NOX4) were validated against neurodegenerative models. Lower biomarker levels were correlated with higher CCDR scores, indicating cognitive decline. Machine-learning analysis revealed the highest predictive accuracy when analyzing the combination of RBP4 and NOX4 using the support vector machine algorithm and confirmed potential diagnostic biomarkers. These results suggest that blood-based biomarkers can notably improve CCDS early detection and treatment, with implications for neurodegenerative disease management in both animals and humans.

## 1 Introduction

Global birth rates and increased human lifespans have sparked a trend in companion animal (CA) ownership, leading to concerns regarding long-term healthcare and medical costs for aging animals (1, 2). With noticeable increases in elderly dog populations, canine cognitive dysfunction syndrome (CCDS) has emerged as a common neurodegenerative condition associated with age (3, 4). In aged dogs, this syndrome shares symptoms reminiscent of Alzheimer’s disease (AD), the most prevalent cause of dementia in humans (4–7). CCDS is characterized by pronounced behavioral changes, such as evident spatial disorientation, nuanced changes in social interactions, compromised adherence to established housetraining protocols, and clear shifts in circadian rhythms and overall activity levels (8, 9). These behavioral alterations, exacerbated by a decline in memory functionality and learning ability (10–12), markedly distress CA owners and present considerable challenges for veterinarians tasked with treating these animals. Senior dogs are particularly vulnerable to CCDS; however, an objective diagnosis and treatment are still lacking. The onset of CCDS often goes unnoticed by CA owners as they frequently overlook early behavioral changes in their dogs (4). Typically, canines aged >7 years begin to display progressive behavioral and cognitive alterations associated with CCDS (8, 13) with the likelihood of developing CCDS increasing considerably with age (10, 12, 14, 15). By the time most senior dogs are diagnosed with CCDS, the condition is usually considerably advanced (4, 13, 16).

In clinical settings, the canine cognitive dysfunction rating (CCDR) is commonly used to identify cognitive deterioration in aging dogs (17); however, existing rating scales for CCDS have practical limitations. The assessment criteria of these scales tend to measure the rate of cognitive decline or frequency of unusual behaviors, potentially lacking the precision required to detect early cognitive changes indicative of CCDS. Identifying these deficiencies at an early stage greatly improves the chances of successful treatment (15). Online or telephone evaluations are susceptible to subjective interpretations by CA owners, possibly leading to over- or underestimation of disease severity (10, 12, 14). Current diagnostic methods for CCDS involve physical and neurological examinations, blood tests (such as serum analysis and complete blood cell count) to identify other conditions with similar symptoms, and the completion of CCDS screening questionnaires by owners (18). Unfortunately, comprehensive clinical tools to evaluate cognitive function in elderly dogs are lacking. While advanced techniques, such as MRI, are ideal for identifying neurological issues and evaluating cognitive deficits, the costs and need for sedation often make veterinary neurologists rely on neurological assessments instead (18). Given the anatomical complexities of the brain, it is difficult to assess progressive pathological shifts directly. However, there is a considerable need for diagnostic procedures based on objective findings from readily available animal biological specimens in clinical settings. Hence, biomarker analysis of the blood and cerebrospinal fluid is expected to emerge as the primary diagnostic method for CCDS (3, 12, 19). However, most CA owners prefer to collect peripheral blood samples over cerebrospinal fluid samples. Although the history of research on CCDS diagnosis using blood analysis is relatively short, recent efforts to identify valid biomarkers have been notable (3, 4, 20–23). Intriguingly, unlike humans, senior dogs with CCDS exhibited negligible Aβ_1-42_ levels and minimal amyloid accumulation in brain tissue (24, 25). Therefore, there is a growing demand for promising alternative biomarkers to detect CCDS. However, proteomic analysis of canine blood using commercially available antibodies presents a notable challenge. Given that most research tools are designed for laboratory animals, there is a considerable limitation to advancing the development of canine biomarkers. Therefore, our selection of biomarkers encompassed those previously validated by our research group (26, 27), along with common biomarkers identified within the proteome array present in the peripheral blood of APP/PS1 mice, a standard model for AD, and the MPTP-induced Parkinson’s disease (PD) model. Using blood analysis, we identified three early biomarkers of CCDS. The biomarkers under study, including retinol-binding protein 4 (RBP4), C-X-C-motif chemokine ligand 10 (CXCL10), and a marker previously identified by our team (NADPH oxidase 4, NOX4), were rigorously validated (26, 27). While numerous studies have suggested the potential of these biomarkers as indicators of nervous system function (28–35), there has been no endeavor to analyze and interpret the results derived from both factors collectively. Comparative proteomic analysis of Alzheimer’s and Parkinson’s disease models in mice and subsequent enzyme-linked immunosorbent assay (ELISA) evaluations underpinned their potential as reliable early indicators of CCDS. Additionally, a machine-learning framework applied to the dataset not only confirmed the robustness of these biomarkers but also their predictive power in clinical applications.

This paper details the methodology and findings of this novel approach with the intention of substantiating blood-based biomarkers as indispensable tools for early CCDS detection. We anticipate that the insights gained from this research will not only enhance CCDS management in dogs, but also offer a translatable framework for addressing human neurodegenerative diseases, thereby enriching the discourse on comparative medicine.

## 2 Materials and methods

### 2.1 Animal study

#### 2.1.1 APP/PS1 mouse Alzheimer’s disease

Serum samples from APP/PS1 transgenic mice were obtained from Laboratory Animal Resources Bank (LAREB, Daegu, Korea) at the National Institute of Food and Drug Safety Evaluation. All experiments were conducted with the approval of the Daegu Gyeongbuk Medical Innovation Foundation (approval number: DGMIF 21111602–00).

#### 2.1.2 MPTP-induced mouse Parkinson’s disease

C57BL/6 J mice (*n* = 20; 7–8 week-old males) were used for the experiments. MPTP (1-methyl-4-phenyl-1,2,3,6-tetrahydropyridine, *n* = 10) was intraperitoneally administered (S47312; Selleck Chemical, Houston, TX, United States) dissolved in 0.9% saline at a dose of 30 mg/kg/day for 30 consecutive days. All experiments were conducted with the approval of the Institutional Animal Care and Use Committee (IACUC) of Soonchunhyang University (approval number: SCH23-0043).

#### 2.1.3 Canine sample acquisition

Blood samples representing 85 canines were collected by partnering with veterinary clinics and requesting that iamdt Co., Ltd. collect the samples with the consent of the CA owners. Blood samples, collected from healthy dogs and dogs with documented cognitive impairment using CCDR scores, were placed in EDTA anticoagulant tubes and separated into plasma and blood cells after centrifugation. Within 1 h of collection, the plasma was transferred to cryogenic tubes and frozen at −80°C until delivery from the veterinary clinics to Soonchunhyang University. All the blood samples used here were reviewed and approved by the IACUC of Soonchunhyang University (approval number: SCH23-0069). Additionally, blood samples from beagle dogs (two aged 5 months and one aged 96 months) that had not undergone any surgical or chemical treatment were obtained from LAREB. The experiments were conducted with the approval of the Daegu Gyeongbuk Medical Innovation Foundation (approval numbers: DGMIF-21071203-00 for the beagle dogs aged 5 months and KMEDI-22071501-00 for the one aged 96 months).

#### 2.1.4 Canine experimental groups

The animals were classified into four groups: normal; mild cognitive impairment (MCI), severe cognitive impairment (SCI), and CCDS. The grouping of categories MCI, SCI, and CCDS is based on the CCDR (12). Initially comprising 13 behavioral items, this scale was refined to 10 questions by excluding three questions that were commonly found to be challenging to answer (Table 1). The cumulative scores range from 0 to 60, with scores between 25 to 35 indicating MCI and scores >36 indicating SCI. Additionally, a CCDS group was incorporated into our classification, encompassing both MCI and SCI.

**Table 1:** The canine cognitive dysfunction rating (CCDR) scale.

CCDR scale		Score				
		1	2	3	4	5
1.How often does your dog pace up and down, walk in circles and/or wander with no direction or purpose		Never	Once a month	Once a week	Once a day	>Once a day
2.How often does your dog stare blankly at the walls or floor?		Never	Once a month	Once a week	Once a day	>Once a day
3.How often does your dog get stuck behind objects and is unable to get around?		Never	Once a month	Once a week	Once a day	>Once a day
4.How often does your dog fail to recognize familiar people or pets?		Never	Once a month	Once a week	Once a day	>Once a day
5.How often does your dog walk into walls or doors?		Never	Once a month	Once a week	Once a day	>Once a day
6.How often does your dog walk away while, or avoid, being patted?		Never	Once a month	Once a week	Once a day	>Once a day
7.Compared with 6 months ago, does your dog now pace up and down, walk in circles and/or wander with no direction or purpose?		Much less	Slightly less	The same	Slightly more	Much more
8.Compared with 6 months ago, does your dog now stare blankly at the walls or floor?		Much less	Slightly less	The same	Slightly more	Much more
9.Compared with 6 months ago, does your dog urinate or defecate in an area it has previously kept clean? (if your dog has never house-soiled, tick ‘the same’)		Much less	Slightly less	The same	Slightly more	Much more
10.Compared with 6 months ago, does your dog fail to recognize familiar people or pets? (Multiply by 3)		Much less	Slightly less	The same	Slightly more	Much more
**Total**		12–24 = Normal 25–35 = MCI^1^ >36 = SCI^2^				

^1^ Mild cognitive impairment, ^2^ Severe cognitive impairment.

### 2.2 Proteome profiler arrays

The cytokines, chemokines, and growth factors present in the mouse serum from both the control and disease groups (AD or PD) were semi-quantitatively evaluated using the Proteome Profiler Mouse XL Cytokine Array Kit (ARY028; R&D Systems, Minneapolis, MN, United States). Serum samples were processed by incubation on nitrocellulose membranes, pre-spotted with capture antibodies in strict adherence to the manufacturer’s instructions. Next, each membrane was treated with a biotinylated antibody cocktail, and subsequently with streptavidin bound to horseradish peroxidase (HRP). HRP luminescence was developed and positive signals were captured using a Chemi Reagent Mix on a light-sensitive X-ray film with exposure times ranging from 1 to 10 min. Quantification of the fold changes involved analysis of the expression levels of each spot on the membrane using HLImage++ Software (v25.0.0r, Western Vision Software, Salt Lake City, UT, United States), and the intensities were compared against the mean values of the control samples.

### 2.3 Antibodies

The following antibodies were used: monoclonal mouse anti-RBP4 antibody (orb751184, Biorbyt, Berkeley, CA, United States); polyclonal rabbit anti-CXCL10 antibody (abx104024, Abbexa, Cambridge, United Kingdom); polyclonal rabbit anti-NOX4 antibody (NB110-58849, Novusbio, Centennial, CO, United States); polyclonal rabbit anti-transferrin antibody (NBP1-97472, Novusbio, Centennial, CO, United States); peroxidase labeled horse anti-mouse IgG (H + L) (7076P2, Cell Signaling Technology, Danvers, MA, United States); peroxidase labeled goat anti-rabbit IgG (H + L) (PI-1000, Vector Laboratories, Burlingame, CA, United States).

### 2.4 Immunoblot

Canine plasma was homogenized in RIPA buffer (R0278, Sigma-Aldrich, St. Louis, MO, United States) and augmented with a phosphatase inhibitor (P3200, GenDEPOT, Barker, TX, United States). To ensure precise analysis of plasma components, a Minute™ albumin depletion reagent (WA-013, Invent Biotechnologies, Plymouth, MN, United States) was employed, effectively removing albumin. The efficacy of this depletion was confirmed using SDS-PAGE and Coomassie Brilliant Blue staining (CR2006, Biosesang, Yongin, Korea). Protein concentrations in albumin-reduced plasma were quantified using a BCA assay kit (21,071, iNtRON Biotechnology, Seongnam, Korea). Following SDS-PAGE on a 10–15% tris-glycine gel, the proteins were transferred onto a PVDF membrane (10,600,023, GE Healthcare, Freiburg, Germany). Membranes were blocked using 5% bovine serum albumin (BSA, SM-BOV, GeneAll Biotechnology, Seoul, Korea) in 1X TBS-T (10X TBS with Tween 20, TR2007, Biosesang, Yongin, Korea). Primary antibodies—specifically monoclonal mouse anti-RBP4, polyclonal rabbit anti-CXCL10, and polyclonal rabbit anti-NOX4—were incubated overnight at 4°C in 1% BSA in TBS-T. HRP-conjugated secondary antibodies, horse anti-mouse, and goat anti-rabbit were then applied for 2 h at ambient temperature. Detection of target proteins was facilitated by ECL western blotting detection reagents, with signals visualized using a chemiluminescence bioimaging instrument (CELLGENTEK, Daejeon-si, Korea). Analytical assessment was performed using the ImageJ software v1.52t (Bethesda, MD, United States).

### 2.5 Enzyme-linked immunosorbent assay (ELISA) for the diagnosis

Blood samples were analyzed for RBP4 (MBS739348), CXCL10 (MBS747479), NOX4 (MBS737351), P-Tau (MBS7230007), and NfL (MBS7231454) using ELISA kits from MyBioSource (San Diego, CA, United States). Each well of a plate, pre-coated with specific detection antibodies from the respective kits, received canine plasma containing RBP4-, CXCL10-, NOX4-, P-Tau-, and NfL-HRP-conjugated antibodies. The reactions were performed at 37°C for 1 h. Following incubation, the plasma and antibodies were discarded, and each well was thoroughly washed with washing buffer. Subsequently, a substrate solution was dispensed into each well, and the plate incubated again at 37°C for 15 min under light-protective conditions. Upon the addition of the stop solution, a colorimetric change from blue to yellow was observed, and the optical densities of the resulting solutions were measured at 450 nm using a microplate reader.

### 2.6 Machine learning for CCDS speculation

Here, we analyzed the CCDS classification using proposed biomarkers for machine learning, for which we utilized Python (v3.6.13)‘s scikit-learn (v0.24.2). In the machine-learning classification task, three comparisons were made: normal vs. MCI, normal vs. SCI, and normal vs. CCDS with the numbers of samples used in these classifications being 50, 46, and 85, respectively. The machine-learning model was trained and evaluated through 10 repeated experiments. Of the total data, 70% were used as training data and the remaining 30% as test data. The machine-learning model utilized various algorithms, including support vector machines (36), extra trees (37), random forests (38), gradient boosting (39), bagging (40), AdaBoost (41), and XGBoost (42), all of which provided by Scikit-Learn. Performance was measured using metrics such as Area Under the Curve (AUC), Accuracy, and the F1 Score.

### 2.7 Statistics

All statistical analyses were performed using Prism 10 (GraphPad Software Inc., San Diego, CA, United States). Data are presented as mean ± standard error of the mean (SEM). Statistical analyses were performed using a Student’s two-tailed t-test to compare the two groups. For multiple group comparisons, an analysis of variance (ANOVA) with *post hoc* comparisons was performed using Tukey’s multiple comparison test. *p* values (^*^ *p* < 0.05; ^**^ *p* < 0.01; ^***^ *p* < 0.001; and ^****^ *p* < 0.0001) were considered statistically significant.

## 3 Results

### 3.1 Novel biomarker screening in AD and PD models

**Figure 1:**
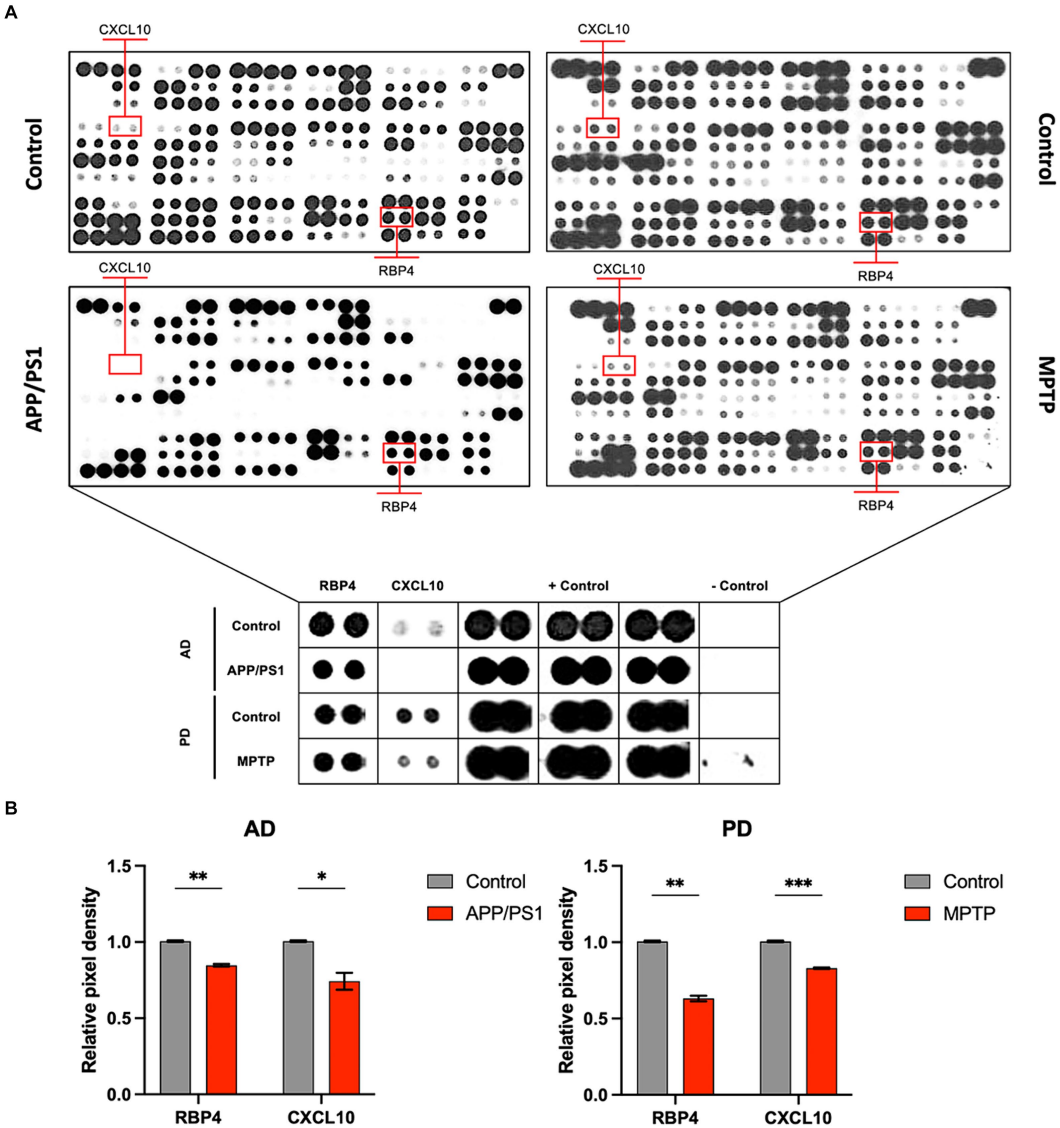
Protein profiling of the serum from APP/PS1 mice and MPTP-induced mice for disease model animals was obtained by performing proteome array analysis. **(A)** Images of array spots on the membrane are proteome array for immune blot, including 111 cytokine, chemokine, and growth factor types. The red rectangle indicates RBP4 and CXCL10 showing expression levels in the APP/PS1 and MPTP-induced mice. Magnifying indicates an enlarged image of RBP4 and CXCL10 in the square below each image. Array spots were analyzed according to the manufacturer’s instructions. **(B)** Quantifying RBP4 and CXCL10 levels in control and disease model animals from serum. Data are mean ± standard error of the mean (SEM). ^*^, *p* < 0.05; ^**^, *p* < 0.01; ^***^, *p* < 0.001 vs. control by a Student’s two-tailed t-test. AD: Alzheimer’s disease; PD: Parkinson’s disease.

Our study involved screening for new biomarkers within the proteomic profiles of blood samples from AD and PD mouse models as well as from normal animals (Figure 1A). Our results indicated that among the 111 inflammatory cytokine markers analyzed, RBP4 and CXCL10 showed significant differential expression in both AD and PD models. These findings suggest that RBP4 and CXCL10 in the blood could serve as reliable indicators of neurodegenerative diseases (Figure 1B).

### 3.2 RBP4, CXCL10, and NOX4 are decreased in the plasma of canine with cognitive decline

#### 3.2.1 General details of the canines used

The general details of the animals used in this study are shown in Table 2.

**Table 2:** Baseline characteristics of all canines in the study.

Groups	Normal	MCI^1^	SCI^2^	CCDS^3^
N = (%)	37 (44)	25 (29)	23 (27)	48 (56)
Age, y (Mean ± SD)	7 ± 3.5	12 ± 3.0	15 ± 2.7	14 ± 3.3
Males, %	16 (43)	14 (56)	11 (48)	25 (52)
CCDR score (Mean ± SD)	24 ± 0.0	30 ± 4.0	49 ± 7.5	40 ± 11.4

^1^ Mild cognitive impairment, ^2^ Severe cognitive impairment, ^3^ Canine cognitive dysfunction syndrome.

#### 3.2.2 Validation of the usefulness of selected biomarkers expressed in canine plasma

**Figure 2:**
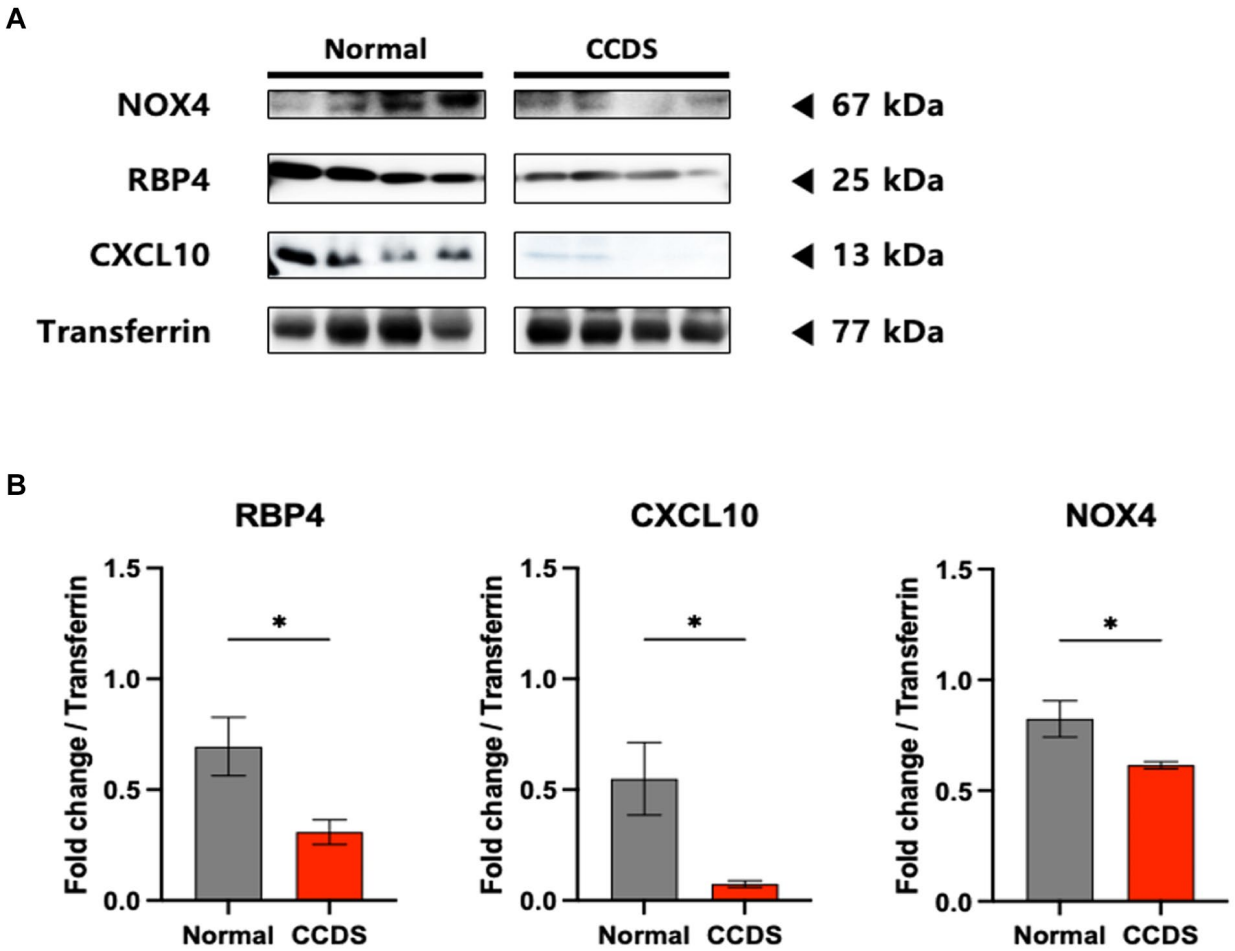
RBP4, CXCL10, and NOX4 protein levels in dogs of normal and CCDS groups. **(A)** Representative immunoblot analysis of RBP4, CXCL10, and NOX4 expression in CCDS groups compared to normal groups. **(B)** Quantifying RBP4, CXCL10, and NOX4 levels in normal and CCDS groups from plasma. Data are mean ± SEM. ^*^, *p* < 0.05 vs. normal by a Student’s two-tailed *t*-test.

To validate the observed results for RBP4 and CXCL10 in the disease animal model, we conducted western blot analysis (Figure 2A). Moreover, we investigated the association between the expression of NOX4—a key molecule contributing to the progression of AD and PD in the brain as identified in previous studies—and the expression of RBP4 and CXCL10 in canines. We identified a significantly lower expression of RBP4, CXCL10, and NOX4 in the plasma of CCDS canines than in normal canines, consistent with the results obtained from APP/PS1 and MPTP-induced mice (Figure 2B).

#### 3.2.3 Biomarker expression measurements in canine blood collected from outpatients using ELISA analysis

**Figure 3:**
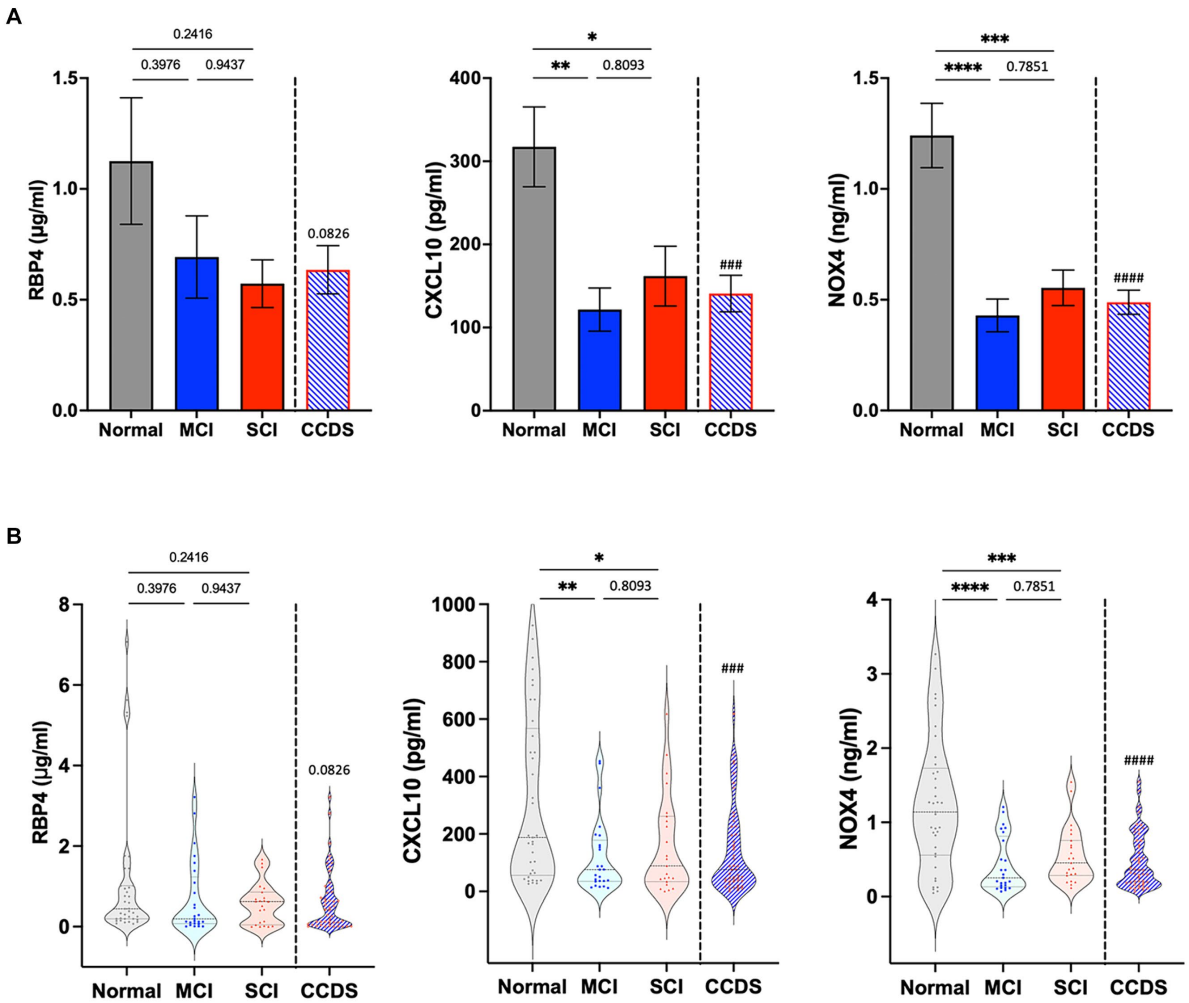
RBP4, CXCL10, and NOX4 levels were measured in the plasma from animal groups diagnosed with mild cognitive impairment (MCI) and severe cognitive impairment (SCI) based on CCDR scores. CCDS represents a group inclusive of both MCI and SCI, separated by a dotted line. **(A)** The bar graphs represent the quantification of RBP4, CXCL10, and NOX4 ELISA levels in each group. Data are mean ± SEM. **(B)** Representative violin plot graphs of the distribution of biomarker levels in each group. The dot in the graph revealed the distribution of individual samples, and the lines in the violin shape represent quartiles and medians. ^*^, *p* < 0.05; ^**^, *p* < 0.01; ^***^, *p* < 0.001; ^****^, *p* < 0.0001 vs. each group by a one-way ANOVA. ^###^, *p* < 0.001; ^####^, *p* < 0.0001 vs. normal by a Student’s two-tailed *t*-test.

We confirmed the utility of the biomarkers by performing ELISA with additional samples (Figure 3). Similarly, decreased expression of RBP4, CXCL10, and NOX4 was observed in the plasma of CCDS dogs compared to normal dogs. Although the *p* value for RBP4 was not significant, a declining trend was noted in the concentrations between normal and CCDS dogs. Notably, in comparison to the normal group, significant decreases in CXCL10 and NOX4 were observed in the MCI and SCI groups, respectively. Likewise, when compared to the combined MCI and SCI group (CCDS group), the measurement values of normal animals and statistical significance were also established. This observation is consistent with the results found in mouse models of the disease.

### 3.3 Machine learning for the CCDS classification

This study applied machine-learning algorithms to evaluate the combination of biomarkers that would most effectively determine a dog’s condition (normal, MCI, SCI, and CCDS). Changing the number of samples used in machine learning can notably affect the results. Therefore, the analysis was performed on an equal number of randomly selected samples in order to equalize the number of normal samples in the analysis group. Ten randomly repeated experiments were performed using seven classification algorithms, with 70% of the total data used as training data in each experiment.

#### 3.3.1 Identification of correlations between variables

**Figure 4:**
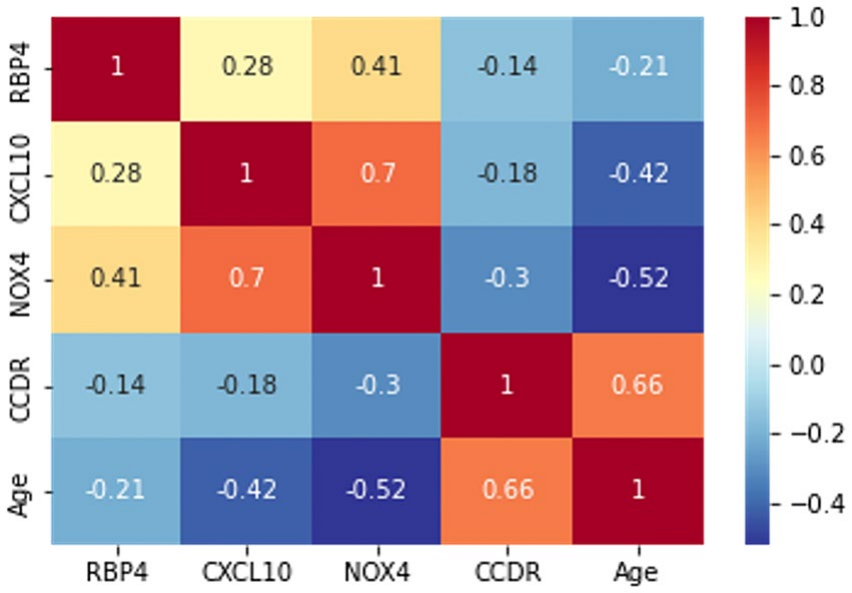
The correlation heatmap manifested the correlation between five variables: RBP4, CXCL10, NOX4, CCDR, and age. The color of each cell indicates a high correlation in red and a low correlation in blue, with the darker color indicating the strength of the correlation.

Prior to the development of the machine-learning model, a correlation heatmap was generated to assess the relationships between the variables using all 85 samples (Figure 4). The variables analyzed included RBP4, CXCL10, NOX4, CCDR, and age. Higher and lower correlations are depicted in red and blue, respectively. Notably, the correlation coefficient between CCDR and age was 0.66, indicating an increasing trend in the CCDR scores with advancing age. A significant correlation (*r* = 0.7) was observed between CXCL10 and NOX4. Furthermore, the results for RBP4, CXCL10, and NOX4 appeared to be inversely proportional to the CCDR scores, with their levels diminishing as CCDR scores increased.

#### 3.3.2 Normal and MCI state prediction

**Figure 5:**
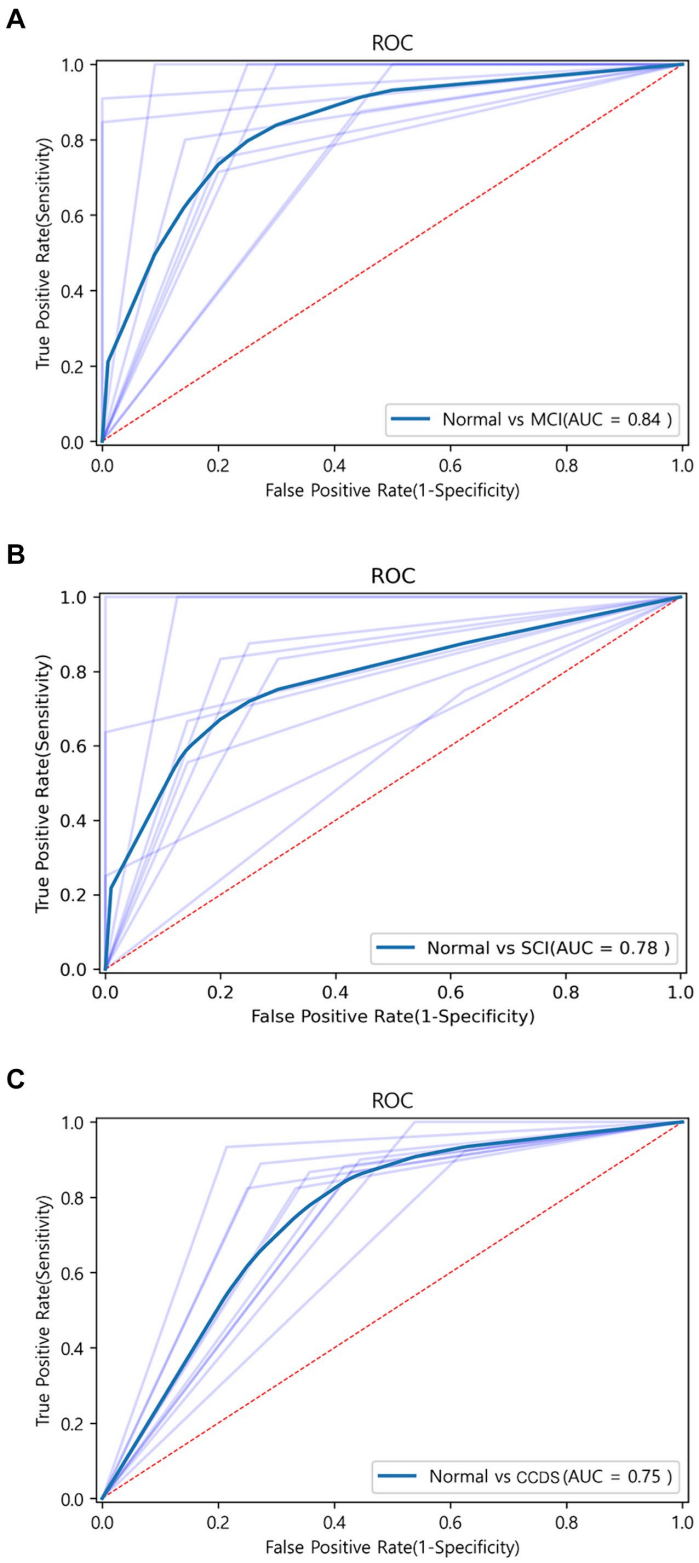
ROC curve graph between each group obtained through machine learning. **(A)** ROC curve for the combination of RBP4 and NOX4 between normal and MCI using the SVM algorithm. AUC is shown as 0.83. **(B)** ROC curve for the combination of RBP4, CXCL10, and NOX4 between normal and SCI using the Extra Tree algorithm. AUC is shown as 0.78. **(C)** ROC curve for the combination of RBP4 and NOX4 between normal and CCDS using the SVM algorithm. AUC is shown as 0.75.

To assess the discriminative ability between normal and MCI, we randomly selected an equal number of normal samples for analysis, matching the 25 MCI samples. Among the biomarker combinations listed in Table 3, the RBP4 and NOX4 combination yielded the highest prediction results, with an F1 score of 0.84. However, the other three combinations resulted in an F1 score of 0.77 (RBP4 + CXCL10 & CXCL10 + NOX4) or 0.73 (RBP4 + CXCL10 + NOX4). In the comparative analysis between normal and MCI samples, the gene combination of RBP4 and NOX4 emerged as the most effective among all combinations (Table 3). Utilization of the receiver operating characteristic (ROC) curve for predicting favorable outcomes in the normal and MCI states was based on animal plasma analysis (Figure 5A).

**Table 3:** Machine learning results summary for the normal and MCI.

Feature	Algorithm	AUC	ACC	Sensitivity	Specificity	Precision	F1
RBP4, CXCL10	Random Forest	0.77	0.76	0.77	0.77	0.78	0.77
RBP4, NOX4	SVM^1^	0.84	0.83	0.89	0.79	0.82	0.84
CXCL10, NOX4	Extra Tree	0.78	0.78	0.83	0.74	0.74	0.77
RBP4, CXCL10, NOX4	SVM^1^	0.64	0.66	0.91	0.38	0.61	0.73

^1^ Support vector machine.

#### 3.3.3 Normal and SCI state prediction

When comparing the results of the three combinations formed by pairing two genes, that of RBP4 and NOX4 exhibited the highest performance (F1 score of 0.73), while that of RBP4 and CXCL10 yielded the lowest result (F1 score of 0.59) (Table 4). Additionally, when all three genes were used, the Extra Tree showed the best analysis result among the seven algorithms (F1 score of 0.75) (Table 4). Despite the small sample size, the use of a combination of the three genes proved to be the most effective set for distinguishing between healthy and SCI patients. Utilization of the ROC curve for predicting favorable outcomes in the normal and SCI states was based on animal plasma analysis (Figure 5B).

**Table 4:** Machine learning results summary for the normal and SCI.

Feature	Algorithm	AUC	ACC	Sensitivity	Specificity	Precision	F1
RBP4, CXCL10	Bagging	0.59	0.61	0.55	0.64	0.53	0.59
RBP4, NOX4	SVM^1^	0.62	0.64	0.92	0.33	0.63	0.73
CXCL10, NOX4	Bagging	0.70	0.69	0.72	0.67	0.72	0.70
RBP4, CXCL10, NOX4	Extra Tree	0.78	0.77	0.73	0.83	0.82	0.75

^1^ Support vector machine.

#### 3.3.4 Normal and CCDS state prediction

For the discrimination between normal and CCDS, the combination of RBP4 and NOX4 biomarkers showed the highest performance with an F1 score of 0.81. In contrast, the combination of CXCL10 and NOX4 exhibited the lowest performance, with an F1 score of 0.74 (Table 5). When utilizing all three genes, resulted in an F1 score of 0.79 (Table 5). The comparison analysis between normal and CCDS samples demonstrated that the combination of RBP4 and NOX4 is the most effective set for distinguishing between the two states. The utilization of ROC curves for predicting favorable results in normal and CCDS conditions was based on plasma analysis (Figure 5C).

**Table 5:** Machine learning results summary for the normal and CCDS.

Feature	Algorithm	AUC	ACC	Sensitivity	Specificity	Precision	F1
RBP4, CXCL10	Extra Tree	0.71	0.71	0.75	0.67	0.78	0.75
RBP4, NOX4	SVM^1^	0.75	0.77	0.89	0.61	0.75	0.81
CXCL10, NOX4	XgBoost	0.69	0.69	0.77	0.60	0.74	0.74
RBP4, CXCL10, NOX4	Extra Tree	0.72	0.72	0.77	0.67	0.78	0.79

^1^ Support Vector Machine.

## 4 Discussion

Despite notable progress in veterinary technology and the increased lifespan of CAs owing to heightened awareness among owners (43), advances in diagnostic technologies for unpredictable cognitive disorders still need to be made. The increasing number of geriatric animals has led to an increase in medical expenses which poses a considerable financial and psychological burden when diagnosing and managing cognitive disorders, such as CCDS, is delayed or missed (44). The difficulty of definitively determining cognitive decline in older animals through CCDR evaluation complicates the diagnosis of CCDS (45). CA owners may overlook subtle deteriorations in the condition of their animals, especially when overt symptoms of cognitive dysfunction are absent (4). This oversight can delay the diagnosis and treatment of CCDS. While the CCDR system is functional, relying solely on it and the subjective assessments of owners and veterinarians may not provide a comprehensive evaluation of the cognitive function of the animal in CCDS cases (6, 45, 46). Collectively, if objective indicators are introduced to differentiate between healthy animals and those exhibiting cognitive dysfunction based on CCDR scores, veterinarians can collaborate with CA owners to formulate precise treatment strategies with greater diagnostic certainty. A comprehensive list detailing the health statuses of the animals involved in the experiments is provided in Supplementary Table S1.

As we mentioned in “2.1.4. Canine Experimental Group” in “Materials & Methods,” the CCDR score used in our study differs somewhat from the score intervals for Normal, MCI, and SCI used in CCDR score (12).

When we initiated this study, veterinarians from the hospitals that provided the CCDR questionnaires used by Salvin et al. (12) indicated that specific behavioral items were challenging for dog owners to respond to, specifically items 7 (“How often does your dog have difficulty finding food dropped on the floor?”), 11 (“Compared with 6 months ago, does your dog have difficulty finding food dropped on the floor”), and 13 (“Compared with 6 months ago, is the amount of time your dog spends active?”) (12). The veterinarians expressed concerns that the owners’ recollections might not be sufficiently accurate to provide reliable responses for these items, potentially leading to erroneous scores that could adversely affect the machine-learning analysis. Considering that the CCDR score depends on the owners’ responses, we deemed it prudent to exclude cases where response accuracy might be compromised to ensure a more reliable machine-learning analysis. Consequently, we decided to exclude the scores for these three items from the total CCDR score. As a result, the scoring ranges for Normal, MCI, and SCI appear different from those proposed by Salvin et al. (12), since we omitted the scores for the three specified items from the total CCDR score. It is important to note that this adjustment does not imply any deficiencies in the study by Salvin et al. (12). Instead, it reflects considerations made by veterinarians in the clinics, tailored to the design and specific requirements of our research.

This study explored novel biomarkers using methodologies similar to those used to identify effective biomarkers of classical AD and PD in experimental animals (Figure 1). Our selection of the potential biomarkers RBP4 and CXCL10 deliberately excluded well-documented biomarkers and those encumbered by intellectual property rights (Supplementary Figures S1, S2). We also incorporated NOX4, anticipated as a notable indicator in various neurological disorders (26, 27, 33, 47). We hypothesized that this approach would enhance the ability to effectively distinguish cognitively impaired animals from their healthy counterparts. The ability to differentiate between normal and cognitively impaired animals via blood analysis is expected to provide a substantial opportunity for making early diagnosis of cognitive disorders and delaying the pathological progression through various therapeutic interventions. However, these endeavors have been markedly constrained by the blood–brain barrier and practical limitations in clinical settings (48, 49). Contrary to our initial hypothesis, the three identified biomarkers demonstrated lower expression levels in the blood of both the experimental animals and those with CCDS than in their healthy counterparts. However, our finding of lower expression levels of these biomarkers in CCDS-afflicted animals was initially unexpected, given their previously documented elevated levels in AD and PD (26, 27). Nonetheless, further analysis confirmed this pattern, suggesting that expression levels may vary selectively based on tissue characteristics. Specifically, in PD models, a marked increase in NOX4 expression is not observed in brain regions other than the hippocampus, highlighting the potential for a differential expression depending on the specific pathology and tissues involved (26). RBP4 belongs to the lipocalin family and serves as the primary transporter for hydrophobic retinol, which is also referred to as vitamin A (29). RBP4 is a plasma protein that specifically binds to retinol and acts as its transporter in the circulation (50). This protein is primarily synthesized in the liver before entering circulation (29). Its influence on the body, shaped by protein expression patterns and interactions with receptors, is complex, leading to various hypotheses (29). Apart from the liver, it is present in the retinal pigment epithelium, testes, adipose tissue, muscle tissue, brain, and choroid plexus (29, 51–53). While the precise function of RBP4 in the central nervous system remains unclear (51, 52), studies have shown that RBP4-deficient mice display decreased mobility and anxiety-like behavior, along with neuronal loss and gliosis in the cerebral cortex and hippocampus (29). Furthermore, evidence suggests a reduction in neuroblast proliferation in the subventricular zone (31). Specifically, the RBP binding site is localized in the endothelium surrounding the choroid plexus, enabling substantial transportation of retinol through the blood–brain barrier (51). The CXC chemokine ligand (CXCL10) is believed to have a significant impact on neuroinflammatory conditions (54), potentially affecting neuronal cells and astrocytes (33). As per Bajova et al., persistent CXCL10 stimulation in the culture model triggers ERK1/2, CREB, and NF-kB pathways, leading to enhanced levels of anti-apoptotic BCL-2 proteins and antioxidant enzymes like manganese superoxide dismutase (SOD2), which offer protection against superoxide radicals (33). The findings of this study indicate a potential close correlation between the presence of CXCL10 and its neuroprotective effects. While the efficacy and associated mechanisms of the three newly suggested biomarkers for early detection of CCDS require further exploration, there is an expectation that they could be beneficially applied in neurodegenerative conditions in canines.

Securing blood samples from outpatients required obtaining consent from their guardians, which notably protracted the sample collection timeline. Moreover, concerns regarding the integrity of the stored blood samples imposed restrictions on the duration of the collection period for analysis. Despite these challenges, the samples utilized here yielded high accuracy and prediction using machine-learning algorithms, indicating that various combinations of biomarkers could distinctly differentiate between normal and cognitively impaired dogs. This outcome underscores the potential of these biomarkers in advancing the diagnostic capabilities of CCDS. A study sought to differentiate between healthy individuals and patients by training machine-learning algorithms using brain imaging data from human patients with AD. However, this approach primarily aimed to ascertain the presence of AD in patients based on existing imaging results (55). In essence, it was not an endeavor to proactively identify individuals deviating from the normative range at an early stage but rather a retrospective confirmation of AD in already diagnosed individuals. Taking this into account, it is highly encouraging that the predictive capability for CCDS is effectively enhanced through the identification and combination of novel blood biomarkers. In a comparison of normal versus MCI and normal versus CCDS, the pairing of the biomarkers RBP4 and NOX4 yielded optimal outcomes with the application of support vector machine (SVM) methods.

Conversely, to distinguish between normal and SCI states, the most effective results were obtained using the Extra Tree algorithm, which incorporated the full spectrum of biomarkers. The complete results of the SVM analysis for various biomarker combinations are shown in Supplementary Tables S2–S4. This outcome suggests that the potency of the predictive algorithm can be further refined and improved by acquiring additional samples in future studies, thereby advancing the early detection and management of CCDS. In other words, although our machine-learning analysis was conducted with a limited number of samples, the accuracy and predictive capability achieved are promising. It is anticipated that with additional sample data, the errors in these metrics will diminish progressively, enhancing their reliability as objective indicators in clinical practice. These results are expected to serve as valuable standards for guiding treatment decisions. Historically, there has been considerable skepticism regarding the feasibility of detecting early biomarkers of cognitive impairment through blood, a sentiment prevalent in both human and veterinary medicine. In canine studies, although certain biomarkers have been identified in the blood, their lack of discriminatory power has rendered them impractical for clinical application (3, 4, 18, 20, 21, 56, 57). We are dedicated to identifying viable biomarkers of dementia-related cognitive impairment and to building a substantial understanding of the pathological mechanisms of these biomarkers. Therefore, we did not rely solely on comparing the ELISA measurements of suspected cognitive impairment biomarkers. Instead, we employed advanced machine-learning analysis tools to assess the existence of CCDS for each unique combination of identified biomarkers, thereby enhancing the precision and applicability of our findings in clinical settings. In other words, if we were simply trying to differentiate between MCI and SCI based on the level of a single biomarker, there would be no need to apply machine learning techniques to train on the combination of results from multiple biomarkers. It is important to remember that a high CCDR score does not necessarily imply a proportional decrease in biomarker expression levels in the blood. Thus, we utilize machine learning with multiple biomarkers as variables because it is challenging to distinguish between Normal, MCI, and SCI using a single biomarker.

Our discoveries show great promise as a means of evaluating the cognitive health of elderly animals in clinical environments, potentially improving the well-being of companion animals and their caregivers. Regular monitoring of these biomarkers could act as a valuable indicator for identifying cognitive impairment early on, thereby enabling early diagnosis and intervention. It’s worth noting that in our study, elderly animals were categorized into MCI and SCI groups based on CCDR scores provided by caregivers, rather than by veterinarians’ long-term observations. The assignment of scores in the CCDR could be influenced by unrelated conditions, such as cataracts leading to blindness. This indicates a bidirectional association between sensory impairment and behavioral changes, potentially indicative of CCDS (58). While our proposed new biomarker combination might not exhibit a distinct statistical variance between MCI and SCI, it’s important to recognize the subjective nature of the CCDR score itself. Our goal is to introduce innovative biomarkers that can detect early signs of cognitive decline and offer insights into disease progression. Therefore, gathering additional clinical samples is essential. Moreover, by observing how these biomarkers respond to treatment in animals diagnosed with CCDS, we aim to assess the effectiveness of our biomarkers. This endeavor mirrors our aspiration to expand our dataset and refine our methodology for identifying cognitive impairment in companion animals, while also differentiating between different forms of degenerative brain disorders. This advancement may pave the way for tailoring treatment strategies, allowing veterinarians to provide precise and prompt care to geriatric animals in clinical settings. This advancement will potentially enable the development of more nuanced treatment strategies, empowering veterinarians in clinical practice to provide targeted and timely care to aging animals. Several dogs classified as MCI or SCI were reported not to have been diagnosed with CCDS but instead exhibited neurological conditions such as MUO (including GME and NME), meningitis, and hydrocephalus in Supplementary Table S1. When looking at the data, these disorders may present with forebrain signs, including restlessness and behavioral changes, which are likely to result in higher scores on the CCDR, a scoring system that relies on clinical observations. These disorders can manifest as forebrain signs, including restlessness and behavioral changes, which are likely to result in higher scores on the CCDR—a scoring system that relies on clinical observations. Another point to consider is whether dogs with a history of brain diseases are more predisposed to developing CCDS later in life, a phenomenon already reported about idiopathic epilepsy in dogs (59, 60). Therefore, in the future, more clinical case data and additional biomarkers will be needed to improve the ability to distinguish between other brain diseases and cognitive impairment.

## 5 Conclusion

We are dedicated to identifying biomarkers that can be used to diagnose cognitive impairment early in elderly animals, thereby providing a critical window for appropriate therapeutic intervention. The source of clinical samples is of paramount importance in this quest as the blood–brain barrier poses substantial challenges in detecting prodromal symptoms of cognitive impairment in both humans and animals. Despite these obstacles, we posit that with the aid of this diagnostic tool, companion animals can enjoy prolonged quality of life alongside their owners, who, in turn, may experience reduced psychological distress as a result of mitigating the behavioral changes associated with severe cognitive decline in their pets. We advocate early screening for cognitive impairment through blood tests, leveraging the predictive values derived from CCDR scores. This approach empowers veterinarians to diagnose and initiate treatment strategies for animals that are likely to develop CCDS and to monitor their brain health through regular follow-up testing. The predictive accuracy and sophistication of our model are expected to improve as we refine our methodology and expand our sample size. Given the substantial diagnostic value demonstrated thus far, we are optimistic regarding the clinical applicability of our method. Looking ahead, we envision that the early detection capabilities of companion animals will pave the way for similar advances in human medicine, thereby broadening the scope and impact of our research.

## Data availability statement

The datasets presented in this study can be found in online repositories. The names of the repository/repositories and accession number(s) can be found in the article/Supplementary material.

## Ethics statement

The animal studies were approved by the Institutional Animal Care and Use Committee (IACUC) of Soonchunhyang University. The studies were conducted in accordance with the local legislation and institutional requirements. Written informed consent was obtained from the owners for the participation of their animals in this study.

## Author contributions

CKi: Methodology, Validation, Writing – original draft. JK: Methodology, Software, Writing – original draft. SYo: Methodology, Software, Writing – original draft. IY: Investigation, Methodology, Writing – original draft. HL: Methodology, Writing – original draft. SS: Methodology, Writing – original draft. DK: Methodology, Writing – original draft. SoK: Methodology, Writing – original draft. S-AK: Conceptualization, Methodology, Writing – review & editing. CKw: Methodology, Writing – original draft. SYi: Conceptualization, Data curation, Funding acquisition, Supervision, Writing – original draft, Writing – review & editing.
