# Supplementary material for: Advancing the early detection of canine cognitive dysfunction syndrome with machine learning-enhanced blood-based biomarkers
Source: Front Vet Sci. 2024 Aug 7;11:1390296. doi: 10.3389/fvets.2024.1390296 (PMC11335684; doi:10.3389/fvets.2024.1390296)
Supplement: Supplementary file 1 [file Data_Sheet_1.pdf]

Supplementary Table 1. Characteristics of the dogs used in this study

No.	Age (years)	Sex	Breed	Main pathology	Medication	CCDR score
**1**	4	F	French Bulldog	Pulmonary hemorrhage		24
**2**	1	M	Bull Terrier			24
**3**	1	M	Bichon Frise			24
**4**	2	F	Bichon Frise			24
**5**	1	M	Maltepoo			24
**6**	1	M	Maltepoo			24
**7**	4	M	Pomeranian			24
**8**	3	F	Pomeranian			24
**9**	4	M	Bichon Frise			24
**10**	4.8	Unknown	Maltese			24
**11**	5	F	Poodle	Gallbladder mucocele, Hip dysplasia		24
**12**	2	M	Mixed	Pancreatitis		24
**13**	2	F	Poodle			24
**14**	8	F	Maltese			24
**15**	8	M	Poodle			24
**16**	8	M	Mixed			24
**17**	12	M	Maltese	MMVD[1] B1		24
**18**	6	F	Labrador Retriever			24
**19**	13	M	Dachshund			24
**20**	9	F	Shih Tzu			24
**21**	7	F	Poodle			24
**22**	11	F	Dachshund			24
**23**	7	F	Maltese	ACL[2] , Bladder stones		24
**24**	7	F	Pomeranian			24
**25**	11	F	Maltese			24
**26**	10	F	Maltese			24
**27**	6	M(N)	Mixed			24
**28**	12	Unknown	Poodle			24
**29**	9	F(S)	Shih Tzu			24
**30**	10.2	F	Spitz	Diabetes	Insulin	24
**31**	8	M	Poodle	Intima Media Thickness	Mycophenolate Mofetil, Prednisolone	24
**32**	6.3	M(N)	Maltese			24
**33**	6	F(S)	Maltese			24
**34**	10.7	M(N)	Italian Greyhound		Levothyroxine	24
**35**	10.3	F(S)	Maltese			24
**36**	8.7	F(S)	Poodle			24
**37**	9	M	Pomeranian			24
**38**	16.5	M(N)	Shih Tzu	CKD[3] stage 2, Gallbladder stone, Pulmonary hypertension		35

**39**	15	M(N)	Shih Tzu	CS[4], Gastric/Intestinal Tumor		34
**40**	9	F(S)	Poodle	Granulomatous Meningoencephalomyelitis	Cyclosporine	35
**41**	14	M	Dachshund	Hypothyroidism	Levothyroxine	30
**42**	9	M	Maltese	Diabetes, CS[4]	Caninsulin, Silymarin, UDCA[14], Trilostane	25
**43**	9	M	Chihuahua			29
**44**	9	M	Bulldog	DIC[5], IVDD[6]		35
**45**	5	F	Pomeranian			33
**46**	9	F	Spitz	Pyometra	Famotidine, Gabapentin, Metronidazole	30
**47**	8	F	Yorkshire Terrier	Diabetes	Neutral Protamine Hagedorn	26
**48**	13	M	Maltese	MMVD[1] stage B1, Blindness		30
**49**	13	F	Dachshund	IVDD[6], MMVD[1]		33
**50**	11.6	F	Maltese	CS[4]		34
**51**	10	M(N)	Poodle	Diabetes	Insulin	26
**52**	12.4	F	Chihuahua	BCS[7] 5-6		25
**53**	12.1	F	Poodle			30
**54**	12	M	Mixed	CS[4]	Trilostane	25
**55**	16	M	Yorkshire Terrier	Bronchitis		32
**56**	16	M	Yorkshire Terrier	CS[4]	Trilostane	35
**57**	16	F	Shih Tzu	Brainstem tumor, IVDD[6]	Phenobarbital, Pimobenclan, Sildenafil, Silymarin, UDCA[14]	26
**58**	13	M	Shih Tzu	Glaucoma, Hypertension	GedaCure	33
**59**	10	F	Maltese	Necrotizing Meningitis, Obesity	KBr, Mycophenolate, Phenobarbital, Zonisamide	26
**60**	14	F	Maltese		Renamezin	26
**61**	13	M	Yorkshire Terrier	CS[4]	Cushing medication, seizure medication, Prednisolone, SAMe	25
**62**	16	M(N)	Shih Tzu	CCDS[8], CVD[9]	Heart Medication	30
**63**	12	F(S)	Maltese	Hydrocephalus	Anticonvulsant	48
**64**	13	M	Maltese			42
**65**	17	M(N)	Shih Tzu	Glaucoma	GedaCure	46
**66**	15	M(N)	Pomeranian	MMVD[1] stage C	Cardiac Medication	43
**67**	17	F(S)	Cocker Spaniel	Glaucoma	GedaCure	56
**68**	11	F(S)	Schnauzer	CKD[3], Dermatophytosis, MMVD[1] stage C	Levothyroxine, Pimobendane, Trilostane	49
**69**	16	M(N)	Shih Tzu	Seizure	Selegiline	48
**70**	9	M(N)	Maltese	MUE[10]	Levetiracetam, MMF[15], Phenobarbital	52
**71**	12	F	Shih Tzu	Periapical Abscess	AMC, Famotidine, Silymarin, UDCA[14]	41
**72**	15	F	Poodle	CCDS[8], CKD[3], Meningitis, Mammary Tumor	Levothyroxine	43
**73**	14	F	Chihuahua	Idiopathic Epilepsy, MMVD[1] stage C	Benazepril, Furosemide, Gabapentin, ISDN, Levetiracetam, Pimobendan, Spironolactone, Theophylline	45
**74**	16	F	Shih Tzu	ACKD[11], CCDS[8], CS[4]		46
**75**	18	Unknown	Unknown	CKD[3], MMVD[1]	Heart Medication, Kidney Medication	56
**76**	15	F	Cocker Spaniel	Hypertension, RCM[12], VSD[13]	Levothyroxine, Prednisolone	60
**77**	14	F	Pinscher		Vinblastine	50
**78**	15	M	Shih Tzu	CKD[3], MMVD[1]	Acetyl cysteine, Amlodipine, Benazepril. Bromhexine, Codeine, Pimobendan, Theophylline	49
**79**	22	M	Yorkshire Terrier	Neuropathic Symptoms		58
**80**	15	M	Maltese			51
**81**	16	F	Shih Tzu	Lymphoma	Marbofloxacin, Prednisolone	39
**82**	15	M(N)	Maltese	CCDS[8]	GedaCure	36
**83**	16	M(N)	Maltese	CCDS[8], Hydrocephalus, IVDD[6]	Selegeline	43
**84**	15	M	Yorkshire Terrier	MUE[10]	Levetiracetam, Prednisolone, Zonisamide	37
**85**	18	F	Yorkshire Terrier	CCDS[8]	Trazodone	52

[1] Mitral Valve Myxomatous Degeneration [2] Torn anterior cruciate ligament [3] Chronic Kidney Disease [4] Cushing's Syndrome [5] Disseminated Intravascular Coagulation [6] Intervertebral Disc Disease [7] Body Condition Score [8] Canine Cognitive Dysfunction Syndrome [9] Cardiovascular Disease [10] Meningoencephalitis Unknown Etiology [11] Acquired Cystic Kidney Disease [12] Restrictive Cardiomyopathy [13] Vestibular System Disorder [14] Ursodeoxycholic Acid [15] Mycophenolate Mofetil

Supplementary Table 2. SVM[1] Results for the Normal and MCI[2]

Feature	AUC	ACC	Sensitivity	Specificity	Precision	F1
RBP4, CXCL10	0.71	0.70	0.85	0.57	0.64	0.72
RBP4, NOX4	0.84	0.83	0.89	0.79	0.82	0.84
CXCL10, NOX4	0.67	0.66	0.94	0.41	0.59	0.72
RBP4, CXCL10, NOX4	0.64	0.66	0.91	0.38	0.61	0.73

[1] Support Vector Machine [2] Mild cognitive impairment

Supplementary Table 3. SVM[1] Results for the Normal and SCI[2]

Feature	AUC	ACC	Sensitivity	Specificity	Precision	F1
RBP4, CXCL10	0.67	0.67	0.93	0.41	0.62	0.74
RBP4, NOX4	0.73	0.71	0.95	0.52	0.64	0.75
CXCL10, NOX4	0.57	0.56	0.78	0.37	0.51	0.59
RBP4, CXCL10, NOX4	0.58	0.56	0.83	0.32	0.52	0.63

[1] Support Vector Machine [2] Severe cognitive impairment

Supplementary Table 4. SVM[1] Results for the Normal and CCDS[2]

Feature	AUC	ACC	Sensitivity	Specificity	Precision	F1
RBP4, CXCL10	0.61	0.66	0.85	0.37	0.67	0.74
RBP4, NOX4	0.75	0.77	0.89	0.61	0.75	0.81
CXCL10, NOX4	0.59	0.66	0.87	0.30	0.65	0.74
RBP4, CXCL10, NOX4	0.58	0.63	0.79	0.37	0.65	0.71

[1] Support Vector Machine [2] Canine cognitive dysfunction syndrome
