# Supplementary figures and images for: Advancing the early detection of canine cognitive dysfunction syndrome with machine learning-enhanced blood-based biomarkers
Source: Front Vet Sci. 2024 Aug 7;11:1390296. doi: 10.3389/fvets.2024.1390296 (PMC11335684; doi:10.3389/fvets.2024.1390296)

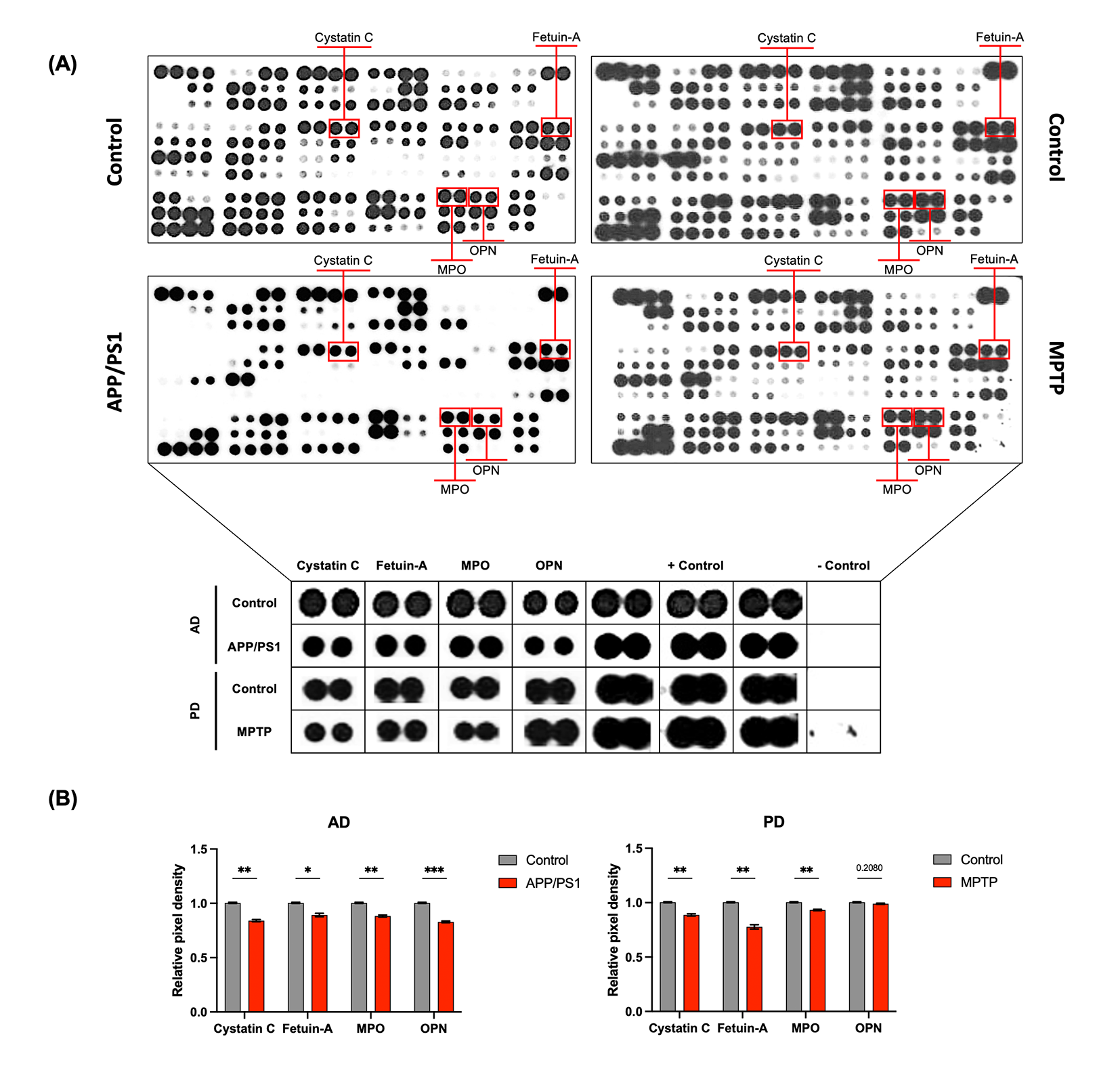

Supplement: Supplementary file 2 [file Image_1.TIF]

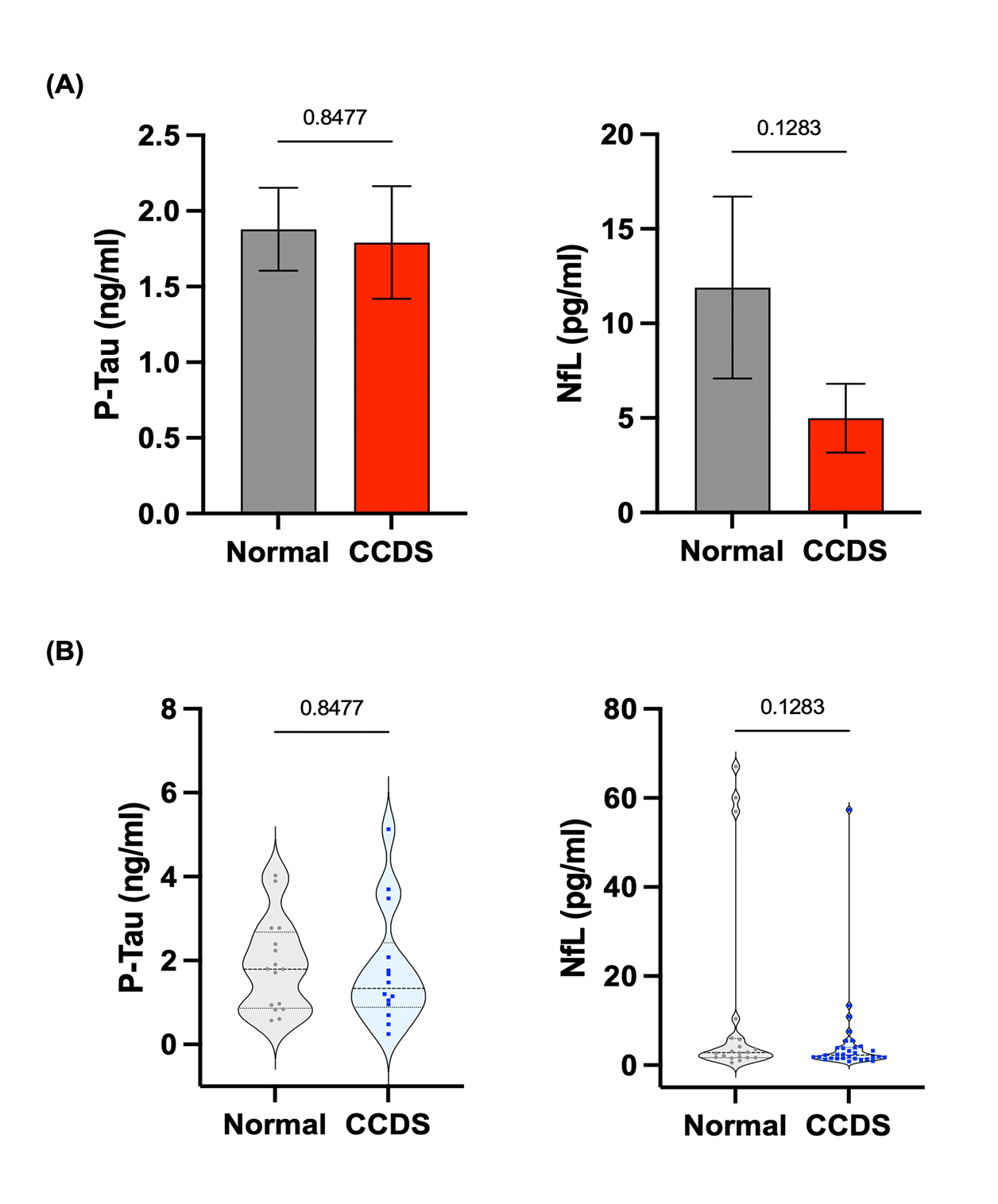

Supplement: Supplementary file 3 [file Image_2.TIF]
